# Immune cytokines as a bridge linking the gut–liver–ovary axis in the pathogenesis of premature ovarian failure

**DOI:** 10.3389/fendo.2026.1758707

**Published:** 2026-03-04

**Authors:** Huimin Xu, Muxi Li, Shouyan Yang, Deyou Jiang

**Affiliations:** 1 College of Basic Medicine, Heilongjiang University of Chinese Medicine, Harbin, Heilongjiang, China; 2 First Clinical Medical College, Heilongjiang University of Chinese Medicine, Harbin, Heilongjiang, China

**Keywords:** bile acid metabolism, gut microbiota, immunity, mechanistic insights, POF

## Abstract

Premature ovarian failure (POF) is a multifactorial disorder characterized by the progressive decline of ovarian function, in which autoimmune factors account for approximately 10%–30% of cases. Accumulating evidence has demonstrated that immune-related mediators, including regulatory T cells (Tregs), interferon-γ (IFN-γ), and T helper 17 (Th17) cells, play pivotal regulatory roles in its initiation and progression. In recent years, the gut–liver axis and its potential mechanistic links with POF have emerged as a research hotspot in this field. Notably, these pathways are closely associated with the expression and functional balance of key immune mediators such as Tregs, IFN-γ, and Th17 cells. Based on the bridging role of immune cytokines between POF and the gut–liver axis, we propose a novel conceptual framework in which immune cytokines serve as a central hub to systematically elucidate the intrinsic connections among POF, gut microbiota dysbiosis, and bile acid metabolism. Furthermore, we highlight the current limitations of existing studies in this area. This perspective may provide a new theoretical framework for understanding the pathogenesis of POF and holds significant scientific value. Importantly, it may also offer novel insights and potential evidence for expanding clinical diagnostic and therapeutic strategies.

## 1 Introduction

Premature ovarian failure (POF) is a clinical syndrome characterized by ovarian insufficiency occurring before the age of 40 years. Its typical features include oligomenorrhea or amenorrhea, elevated follicle-stimulating hormone (FSH) levels, and decreased estrogen concentrations (1). As one of the leading causes of female infertility, POF not only impairs reproductive function but is also associated with multiple systemic complications, including mood disorders, cognitive decline, osteoporosis, and cardiovascular diseases, and it significantly increases the risk of all-cause mortality (2–5). Current studies indicate that its pathogenesis involves multifactorial interactions, with autoimmune factors accounting for approximately 10%–30% of cases (6, 7). Dysregulation of immune cell subsets and cytokines—such as regulatory T cells (Treg), interferon-γ (IFN-γ), and T helper 17 (Th17) cells—has been confirmed to participate in disease progression. In recent years, the gut microbiota and bile acid metabolism have emerged as research hotspots, and their interactions with Treg, IFN-γ, Th17, and related immune mediators are increasingly being elucidated. Based on the bridging role of immune cytokines between POF and gut microecology as well as bile acid metabolism, this article proposes using immune cytokines as a central connecting hub to explore in depth the intrinsic relationship between POF and the gut microbiota, aiming to provide a theoretical foundation and research direction for future microbiota-based interventions and therapeutic strategies in POF.

Autoimmune POF is fundamentally characterized by immune-mediated ovarian attack. It is primarily manifested by infiltration of T cells and macrophages into ovarian tissue or the production of anti-ovarian antibodies (such as anti–zona pellucida antibodies and anti–3β-hydroxysteroid dehydrogenase [3β-HSD] antibodies), leading to the erroneous recognition and destruction of ovarian follicles. The ovaries often exhibit lymphocytic inflammatory infiltration, with CD4^+^ T cells showing a cuff-like distribution surrounding antral follicles. Such patients frequently present with autoimmune polyglandular syndrome (APS), particularly APS type 3 (autoimmune thyroid disease combined with POF), and circulating anti-adrenal or anti-ovarian antibodies can be detected in serum. Their chromosomal karyotype is typically normal, and no pathogenic mutations are identified in genes such as FMR1 or FSHR.

In contrast, non-autoimmune POF (including genetically determined or idiopathic forms) is mainly associated with X chromosome abnormalities, such as Turner mosaicism and FMR1 premutation, or caused by mutations in genes related to follicular development or receptor defects. The ovaries usually exhibit atrophic and fibrotic changes without immune cell infiltration. The most fundamental distinction between the two types is that autoimmune POF originates from abnormal immune attacks on the ovary, whereas non-autoimmune POF results from irreversible defects intrinsic to the follicles or their regulatory genes.

The gut–liver–ovary axis represents a central driver in autoimmune POF. Autoimmune POF is characterized by ovarian autoantibodies, CD4^+^ T cell infiltration, and oophoritis. The gut–liver–ovary axis intervenes through two major pathways. One is the molecular mimicry pathway: studies suggest that specific gut microbiota (such as Chitinophaga and certain Bacteroides species) possess outer membrane proteins structurally similar to ovarian antigens (3β-HSD and zona pellucida proteins). When gut dysbiosis occurs, intestinal immune tolerance may be disrupted, leading to the generation of cross-reactive antibodies. These antibodies reach the ovary via systemic circulation, selectively attack follicles, and ultimately result in follicular depletion. The second is the barrier disruption pathway: abnormal bile acid metabolism (particularly insufficient GDCA/TUDCA) may impair the ILC3–IL-22 signaling pathway, thereby causing defective intestinal mucosal repair and increased intestinal permeability. Microbial products such as lipopolysaccharide (LPS) enter the circulation and activate ovarian macrophages through TLR4, promoting M1 polarization and aggravating local autoimmune injury. In autoimmune POF, regulation of this axis holds therapeutic potential.

Unlike autoimmune POF, non-autoimmune POF is primarily caused by genetic defects, including X chromosome abnormalities (such as Turner mosaicism), FMR1 premutation, and FSHR mutations. These patients have a congenitally insufficient follicular reserve and intrinsically accelerated follicular atresia. Although the gut–liver–ovary axis may prolong the survival of residual follicles by improving the ovarian microenvironment, it cannot reverse follicular loss caused by genetic defects.

When encountering a patient with POF, the first step is not microbiota testing but etiological stratification. Karyotype analysis and screening of FMR1, FSHR, NOBOX, and related genes should be performed to exclude genetic mutations. Subsequently, anti-ovarian antibodies, anti-adrenal antibodies, and anti-thyroid antibodies should be assessed to determine autoimmune involvement. If a genetic mutation is identified, microbiota intervention should be considered only as a strategy to protect residual follicles, without expectations of curative effects. If the condition is autoimmune in nature, the gut–liver–ovary axis should be regarded as a core therapeutic target.

1.Regulatory T cells (Tregs)

In 1995, the research team led by Sakaguchi first identified and characterized regulatory T cells (Regulatory T cells, Tregs), a subset of CD4^+^CD25^+^ T cells possessing potent immunosuppressive functions. In the peripheral blood of both humans and mice, Tregs account for approximately 5%–10% of total CD4^+^ T cells (8). Tregs can be broadly classified into two major subsets: naturally occurring regulatory T cells (nTregs) and inducible regulatory T cells (iTregs). In most biomedical research contexts, CD4^+^CD25^+^ Tregs typically refer to nTregs (9).

Tregs play a central regulatory role in maintaining immune tolerance and preventing autoimmunity by suppressing the activation and proliferation of autoreactive T cells. Their immunosuppressive function is primarily dependent on the transcription factor Foxp3, as well as the secretion of multiple immunomodulatory cytokines, including IL-2, IL-4, IL-10, transforming growth factor-β (TGF-β), and tumor necrosis factor-α (TNF-α). Notably, these molecules are not only essential for fine-tuning immune homeostasis but also participate in critical physiological processes such as embryonic development, tissue repair, and wound healing. A significant reduction in Treg number or functional impairment can disrupt immune tolerance, thereby contributing to the initiation and progression of various autoimmune diseases.

### 1.1 Tregs and the gut microbiota

The gut microecosystem represents the largest and most complex microbial community in the human body. It comprises more than 1,000 bacterial species, with a total population reaching approximately 10¹^4^ microorganisms and a combined mass of up to 1 kg, and is therefore often referred to as the “second genome” of the human body (10). These symbiotic microorganisms not only contribute to the development and maturation of the intestinal mucosal immune system but also promote the production of secretory immunoglobulin A (sIgA) and establish intricate interaction networks with various intestinal immune cells, collectively maintaining intestinal microenvironmental homeostasis.

Accumulating evidence indicates that the gut microbiota and its metabolites play pivotal roles in regulating Treg proliferation and differentiation. Dysbiosis is frequently accompanied by a reduction in Treg abundance and functional impairment, as well as decreased synthesis of beneficial metabolites such as short-chain fatty acids (SCFAs) (11). Smith et al. (12) first reported that SCFAs produced by commensal bacterial fermentation of dietary fiber significantly increased the number of colonic Tregs and enhanced their immunoregulatory capacity. A 2019 study further demonstrated that the compound DAPH promoted Treg differentiation and suppressed the pro-inflammatory activity of Th17 cells by enriching SCFA-producing bacteria, thereby alleviating experimental colitis and improving intestinal barrier function and immune homeostasis (13).

In addition, Atarashi et al. (14) found that Clostridium clusters IV and XIVa increased local transforming growth factor-β (TGF-β) levels and promoted Foxp3 expression while inducing Th17 differentiation, ultimately enhancing Treg-mediated anti-inflammatory responses. Based on this mechanism, fecal microbiota transplantation (FMT), which reshapes the intestinal microbial structure and restores the Treg/Th17 balance, has been clinically applied to ameliorate inflammatory bowel diseases such as ulcerative colitis (15).

### 1.2 Tregs and bile acid metabolism

Bile acids are amphipathic molecules synthesized in the liver from cholesterol, containing both hydrophilic and hydrophobic moieties. As the principal organic components of bile, their fundamental physiological function is to emulsify dietary lipids and facilitate the absorption of lipids and fat-soluble vitamins. The core feature of bile acid metabolism is the highly efficient enterohepatic circulation.

In the liver, cholesterol is converted into primary bile acids, mainly cholic acid (CA) and chenodeoxycholic acid (CDCA). These primary bile acids are subsequently conjugated with glycine or taurine to form more water-soluble and stable conjugated bile acids, such as glycocholic acid (GCA) and taurocholic acid (TCA), thereby adapting to the gastrointestinal environment (16). Upon entering the intestine, conjugated bile acids are hydrolyzed by bile salt hydrolases (BSHs) secreted by gut microbes (e.g., Clostridium and other anaerobes), releasing free bile acids. Furthermore, certain primary bile acids are metabolized by intestinal microbiota into secondary bile acids, including deoxycholic acid (DCA) and lithocholic acid (LCA). Approximately 95% of bile acids (both primary and secondary forms) are actively reabsorbed in the terminal ileum, transported back to the liver via the portal circulation, and reprocessed by hepatocytes for resecretion into bile, thereby achieving efficient recycling (17, 18).

Beyond their classical role in lipid digestion, bile acids are now recognized as key signaling molecules with diverse physiological regulatory functions. Indeed, bile acids mediate a sophisticated inter-organ communication network encompassing hepatic synthesis, microbial modification in the gut, and systemic signaling, and play a central role in maintaining metabolic homeostasis (19).

Bile acids and regulatory T cells form a mutually reinforcing immune homeostatic axis. Through activation of specific receptor-mediated signaling pathways, bile acids directly or indirectly promote Treg differentiation and functional stability, thereby enhancing immune tolerance (20). Conversely, Tregs maintain systemic immune homeostasis and provide a stable physiological environment for bile acid metabolism, forming a bidirectional regulatory loop.

Farnesoid X receptor (FXR) signaling suppresses pro-inflammatory pathways such as NF-κB and reduces the production of inflammatory cytokines, including IL-6 and TNF-α, in the intestine (17). An anti-inflammatory intestinal milieu is more conducive to Treg differentiation and functional maintenance. Moreover, FXR activation preserves intestinal epithelial barrier integrity and reduces the systemic translocation of pathogen-associated molecular patterns such as lipopolysaccharide (LPS), thereby preventing systemic inflammatory activation and creating a favorable microenvironment for Tregs. By fostering an anti-inflammatory and tolerogenic intestinal environment, FXR signaling indirectly supports Treg stability and function (16).

Secondary bile acids, particularly LCA and DCA, bind to Takeda G protein-coupled receptor 5 (TGR5) on antigen-presenting cells (APCs) and macrophages, inhibiting NF-κB signaling, reducing pro-inflammatory cytokine production, and promoting the secretion of the anti-inflammatory cytokine IL-10 (21). This anti-inflammatory APC/macrophage phenotype preferentially induces naïve T cells to differentiate into Tregs rather than pro-inflammatory Th17 cells during antigen presentation. TGR5 signaling is also present in intestinal enteroendocrine L cells, where its activation stimulates glucagon-like peptide-1 (GLP-1) secretion, which exerts anti-inflammatory effects and may indirectly influence Treg responses.

Intestinal dendritic cells express retinaldehyde dehydrogenase (RALDH), which converts dietary vitamin A into retinoic acid. Bile acids are essential for the absorption of lipids and fat-soluble vitamins, including vitamin A. Some studies suggest that certain bile acids directly upregulate RALDH expression in dendritic cells. Retinoic acid binds to retinoic acid receptors in T cells and promotes Foxp3 transcription, the master transcription factor governing Treg lineage commitment. Therefore, by facilitating retinoic acid generation, bile acids indirectly yet potently drive Treg differentiation (22).

Tregs protect hepatocytes and cholangiocytes from immune-mediated injury by suppressing excessive immune responses (23). In autoimmune liver diseases such as primary sclerosing cholangitis, Treg dysfunction or depletion contributes to bile duct destruction and cholestasis. Chronic hepatic inflammation alters the expression of metabolic genes in the liver. In a pro-inflammatory environment lacking adequate Treg regulation, cytokine-mediated effects may indirectly influence rate-limiting enzymes such as CYP7A1 in hepatocytes, thereby altering bile acid synthesis rate and composition.

Furthermore, Tregs are essential for maintaining intestinal immune tolerance and regulating gut microbial composition. Since gut microbiota are the key mediators converting primary bile acids into secondary bile acids, Tregs can indirectly regulate enterohepatic bile acid circulation and bile acid pool composition by shaping microbial communities (24).

Collectively, bile acids and Tregs form a sophisticated immunometabolic dialogue. Bile acids actively shape an immune-tolerant environment conducive to Treg generation, while Tregs, by maintaining global immune homeostasis, safeguard the proper progression of bile acid metabolism (25).

### 1.3 Tregs and POF

Regulatory T cells (Tregs) comprise multiple subsets, including naturally occurring Tregs (nTregs), inducible Tregs (iTregs), CD8^+^ Tregs, and natural killer T (NKT) cells. Functional abnormalities in these subsets are closely associated with various autoimmune diseases. Among them, Th3-type CD4^+^ regulatory T cells participate in ovarian development throughout its course by secreting transforming growth factor-β (TGF-β), thereby promoting the proliferation and differentiation of granulosa cells and oocytes. As a key signaling molecule, TGF-β belongs to a superfamily that also includes critical reproductive regulators such as follicle-stimulating hormone (FSH) and luteinizing hormone (LH), and it plays a central role in maintaining ovarian endocrine function through the regulation of steroidogenesis.

Recent studies have demonstrated a significant association between Tregs and POF. Various therapeutic strategies may exert beneficial effects on POF by increasing Treg numbers or enhancing their immunoregulatory function. In a mouse model of POF, Yin et al. (26) reported that transplantation of human mesenchymal stem cells (hMSCs) promoted ovarian functional recovery, potentially through modulation of the PI3K/Akt signaling pathway and restoration of the Th17/Tc17 and Th17/Treg balance.

Furthermore, evidence indicates that the traditional Chinese medicine formulation Bushen Huoxue decoction increased the proportion of CD4^+^CD25^+^Foxp3^+^ Tregs in the spleen of autoimmune POF mice, suppressed excessive CD4^+^ T cell activation, and promoted Treg proliferation. This intervention also reduced serum levels of anti–zona pellucida antibodies, IL-10, and IFN-γ, thereby exerting immunomodulatory and ovarian-protective effects (27).

Zhang et al. (28) demonstrated that human amniotic epithelial cells elevated splenic Treg levels in autoimmune POF mice, restored ovarian function, and alleviated local inflammation through paracrine modulation of macrophage activity. In addition, Song et al. (29) reported that combined therapy with human adipose-derived mesenchymal stem cells and estrogen promoted Treg expansion, exerted immunoregulatory effects, and contributed to the improvement of ovarian function.

Collectively, these findings suggest that modulation of Treg-mediated immune tolerance may represent a promising therapeutic avenue in POF.

## 2 Interferon-γ

Interferon-γ (IFN-γ) is primarily produced by activated T cells and natural killer (NK) cells. It is a signature Th1-type cytokine with potent pro-inflammatory properties and the capacity to induce autoimmune responses, playing a central role in both innate and adaptive immunity (30).

### 2.1 IFN-γ and the gut microbiota

Alterations in intestinal microecological balance and exogenous interventions can significantly modulate IFN-γ expression. Infection with Helicobacter hepaticus activates the IFN-γ/p-STAT1 signaling pathway, leading to the formation of a detrimental immune microenvironment. In addition, bacterial translocation–induced small intestinal bacterial overgrowth is frequently associated with elevated expression of IFN-γ, IL-4, IL-17, and mucin-2, thereby promoting inflammatory responses, disrupting intestinal barrier integrity, increasing permeability, and exacerbating hepatic injury (31).

Interventional studies have shown that Lactobacillus reuteri reduces the Th1/Th17 ratio and decreases IFN-γ and IL-17 levels in experimental autoimmune encephalomyelitis models (32). Clinical trials further demonstrate that probiotic supplementation increases serum IFN-γ and intestinal secretory IgA (sIgA) levels in adults with recurrent colds (33). Moreover, infections with Eimeria species and Clostridium perfringens significantly upregulate the mRNA expression of claudin-2, IFN-γ, TLR2, and NOD1 (34).

Notably, certain microbial components—such as Lactobacillus, Escherichia coli, and bacteriophage DNA—can induce IFN-γ responses through TLR9 signaling, thereby promoting phage expansion and aggravating colitis. Studies have shown that ulcerative colitis patients who respond favorably to fecal microbiota transplantation (FMT) exhibit relatively lower intestinal bacteriophage abundance, and mucosal IFN-γ levels positively correlate with bacteriophage numbers (35).

Collectively, the gut microbiota and IFN-γ exert bidirectional regulatory effects. Microbial composition influences IFN-γ secretion, whereas IFN-γ can reciprocally modulate intestinal microecology through immune-mediated mechanisms. For example, specific commensals activate IFN-γ signaling to enhance host defense against Salmonella infection (36). Conversely, IFN-γ participates in immune responses to Vibrio cholerae and its lipopolysaccharide (LPS) by suppressing DMBT1 gene expression in intestinal epithelial cells (37).

### 2.2 IFN-γ and bile acid metabolism

A bidirectional relationship exists between IFN-γ and bile acid metabolism. As a pro-inflammatory cytokine, IFN-γ disrupts and remodels normal bile acid homeostasis, whereas bile acids can suppress IFN-γ production through receptor-mediated signaling pathways, thereby exerting anti-inflammatory effects (38).

IFN-γ primarily impairs bile acid homeostasis by inducing inflammatory responses and altering the expression of key metabolic genes. Through activation of the JAK–STAT1 signaling pathway, IFN-γ suppresses CYP7A1 transcription, thereby modifying bile acid pool composition and influencing lipid digestion, absorption, and cholesterol homeostasis. In addition, hepatocyte membrane transporters responsible for bile acid uptake and efflux—such as bile salt export pump (BSEP) and sodium taurocholate cotransporting polypeptide (NTCP)—may be downregulated in IFN-γ–mediated inflammatory environments. This results in intrahepatic bile acid accumulation, hepatocellular injury, oxidative stress, and inflammation. IFN-γ also induces intestinal epithelial apoptosis and disrupts tight junction integrity (39).

As signaling molecules, bile acids actively modulate immune responses in a manner partially analogous to Treg-mediated regulation (40). Upon TGR5 activation, intracellular cyclic AMP (cAMP) levels increase, leading to inhibition of NLRP3 inflammasome activation and suppression of pro-inflammatory cytokine production (41, 42). This promotes an anti-inflammatory (M2-like) macrophage phenotype, reducing the release of cytokines such as IL-1β and TNF-α, which synergize with IFN-γ in driving inflammation. Indirectly, this attenuates Th1 differentiation and activation—the principal cellular source of IFN-γ.

### 2.3 IFN-γ and POF

IFN-γ plays a key regulatory role in the pathogenesis of POF. Follicular development, maturation, ovulation, and atresia are tightly regulated by a complex cytokine network. Under conditions of immune dysregulation, increased CD4^+^T cell activity leads to excessive IFN-γ production. Elevated IFN-γ signaling induces aberrant expression of major histocompatibility complex class II (MHC II) molecules on ovarian granulosa cells. Such abnormal expression is closely associated with autoimmune responses. Once recognized as “non-self,” these MHC II–expressing granulosa cells trigger immune activation, accelerate follicular atresia, and contribute to ovarian failure (43).

Furthermore, IFN-γ upregulates IL-1βand transforming growth factor-α (TGF-α), promoting B cell proliferation and antibody production. This enhances the activation of cytotoxic T lymphocytes (CTLs), NK cells, and lymphokine-activated killer (LAK) cells, thereby augmenting CD8^+^ T cell cytotoxicity. The resulting immune cascade leads to granulosa cell destruction, follicular structural damage, and antigen-target cell apoptosis, ultimately accelerating excessive follicular atresia and precipitating POF (44).

Therapeutically, transplantation of human placental mesenchymal stem cells (hpMSCs) has emerged as a potential strategy for POF treatment. Experimental studies demonstrate that hpMSC transplantation improves ovarian function in POF model mice, characterized by restoration of serum TGF-βlevels and reduction of IFN-γ concentrations. These findings suggest that hpMSCs may reestablish immune homeostasis and promote ovarian repair by modulating the balance of key cytokines (45).

## 3 T helper 17 cells

Th17 cells differentiate from naïve CD4^+^T cells and are characterized by IL-17 secretion and pro-inflammatory activity. Since their identification in 2005, Th17 cells have been recognized as the third major T helper subset, alongside Th1 and Th2 cells (46, 47). Their discovery challenged the classical Th1/Th2 paradigm and provided new insights into the immunopathogenesis of autoimmune diseases (48).

The differentiation and maintenance of Th17 cells depend on coordinated signaling from cytokines including TGF-β, IL-6, IL-21, and IL-23 (49). Their transcriptional regulation and developmental pathways differ substantially from those of Th1 and Th2 cells (50). Notably, although both Th17 and Tregs require TGF-β signaling for differentiation, they exert opposing immunological functions. Th17 cells promote inflammation and participate in autoimmune pathology, whereas Tregs maintain immune tolerance through immunosuppressive mechanisms. An imbalance in the Th17/Treg ratio is widely regarded as a central immunological hallmark of autoimmune disease (51, 52). This balance is regulated by multiple mechanisms, including T cell receptor (TCR) signaling, co-stimulatory molecules, cytokine milieu, Foxp3 stability, metabolic pathways, and gut microbiota composition. Maintenance of dynamic Th17/Treg equilibrium is therefore critical for preventing immune dysregulation and chronic inflammation.

### 3.1 Th17 cells and the gut microbiota

Studies indicate that the P. UF1 strain isolated from the intestines of breastfed preterm infants attenuates pathogen-induced inflammation and colonic tissue injury. This effect is primarily mediated by IL-10^+^Tregs, which suppress the Th17–Th1 axis and prevent systemic inflammation and tissue damage induced by pathogens such as Listeria monocytogenes (53).

Britton et al. (54) demonstrated that transplantation of gut microbiota from inflammatory bowel disease patients into germ-free mice increased Th17 and Th2 cell frequencies in the small intestine while reducing RORγt^+^Tregs, suggesting that dysbiosis promotes inflammatory immune activation. Čosič et al. (55) further reported a negative correlation between Th17 frequency and Prevotella abundance in the human small intestine, implicating specific microbial imbalances in Th17 expansion and autoimmune susceptibility.

Natural bioactive compounds also modulate Th17/Treg balance via microbial regulation. Portulaca oleracea increases gut microbial diversity, particularly SCFA-producing bacteria, thereby promoting Treg differentiation and suppressing Th17 responses (56). Dachengqi decoction significantly improves microbial composition, increases the abundance of Firmicutes and Actinobacteria, enhances butyrate-producing bacteria, and restores mesenteric lymph node Th17/Treg balance, thereby reestablishing immune homeostasis (57).

### 3.2 Th17 cells and bile acid metabolism

Bile acids suppress Th17 differentiation and function via receptor-mediated signaling, exerting anti-inflammatory effects, whereas Th17-driven inflammation disrupts bile acid metabolic homeostasis (58). Activation of FXR in intestinal cells reduces IL-17 production and inhibits Th17 differentiation. Binding of bile acids to TGR5 elevates intracellular cAMP levels and decreases IL-6 and IL-23 production, thereby inhibiting Th17 differentiation at its source. By modulating the abundance of “pro-Th17” microbial taxa, bile acids can indirectly regulate systemic Th17 levels (59).

Conversely, Th17-mediated inflammation damages hepatocytes and cholangiocytes, leading to bile acid metabolic dysfunction. Altered bile acid pool composition—particularly reduced anti-inflammatory secondary bile acids—weakens suppression of Th17 responses, thereby intensifying inflammation and forming a vicious cycle of “inflammation–bile acid dysregulation–exacerbated inflammation” (60).

### 3.3 Th17 cells and POF

Direct evidence linking Th17 cells to POF remains limited; however, emerging data suggest a potential mechanistic role (61). An important study demonstrated that in a human POF mouse model, human mesenchymal stem cell (hMSC) transplantation activated the PI3K/Akt pathway, corrected Th17/Tc17 and Th17/Treg imbalances, restored immune homeostasis, and promoted ovarian functional reconstruction (62, 63). These findings imply that Th17-mediated immune dysregulation may contribute to POF pathophysiology. Given the limited current evidence, further mechanistic investigation into the role of Th17 cells in POF represents a promising direction for future research.

## 4 The gut–liver–ovary axis

### 4.1 Mechanistic basis of the gut–liver–ovary axis

Recent work by the team of Qiao Jie at Peking University demonstrated that patients with polycystic ovary syndrome (PCOS) exhibit a significant increase in Bacteroides vulgatus in the gut microbiota. The abundance of B. vulgatus positively correlates with circulating androgen levels and negatively correlates with ovulatory function. Fecal microbiota transplantation (FMT) from PCOS patients into germ-free mice successfully recapitulated PCOS-like phenotypes, including polycystic ovarian morphology and estrous cycle disruption. Moreover, transplantation of B. vulgatus alone reproduced similar features, indicating a causal role of this bacterium in PCOS pathogenesis.

Mechanistically, B. vulgatus produces high levels of bile salt hydrolase (BSH), which hydrolyzes conjugated bile acids (e.g., glycine- and taurine-conjugated forms) into free bile acids, thereby reducing biologically active bile acids such as glycodeoxycholic acid (GDCA) and tauroursodeoxycholic acid (TUDCA). GDCA and TUDCA serve as important agonists of the Takeda G protein–coupled receptor 5 (TGR5). Their depletion suppresses the TGR5–GATA3 signaling pathway in group 3 innate lymphoid cells (ILC3s), impairing ILC3-derived interleukin-22 (IL-22) secretion. IL-22 deficiency directly disrupts the ovarian follicular microenvironment, thereby compromising ovarian function.

This “B. vulgatus–BSH–GDCA/TUDCA–ILC3–IL-22–ovary” pathway represents one of the most mechanistically defined and causally validated signaling cascades within gut–reproductive axis research. It integrates gut microbiota, bile acid metabolism, innate immunity, and ovarian physiology, providing novel therapeutic targets for PCOS.

However, translational challenges remain. The cellular distribution of bile acid receptors and IL-22 receptors within the ovary has not been comprehensively mapped. Furthermore, potential interventions—including TUDCA supplementation, BSH-targeted bacteriophages, and IL-22–based therapeutics—have not yet been evaluated in reproductive clinical settings. Despite these limitations, this pathway has been causally validated in PCOS and shows therapeutic promise in autoimmune premature ovarian failure (POF), highlighting the potential of microbiota-centered interventions in reproductive endocrine disorders.

### 4.2 Key signaling pathways of the gut–liver–ovary axis

The introduction of the gut–liver–ovary axis represents a conceptual shift in reproductive endocrinology—from a traditional organ-centered framework toward an integrated metabolic–immune network perspective. These three organs collectively form a coordinated system that transmits both metabolic and immune signals through circulating molecular mediators.

In physiology, an “axis” refers to a coordinated and often bidirectional flow of regulatory information that functionally integrates anatomically distinct organs. The classical hypothalamic–pituitary–ovarian axis operates through hormone gradients and feedback loops. In contrast, the gut, liver, and ovary lack direct anatomical continuity and do not share a dedicated endocrine portal system. Their connection is instead established through the enterohepatic circulation of bile acids.

The enterohepatic circulation serves as the primary structural and functional link among these organs. The liver synthesizes primary bile acids and secretes them into the intestine. Within the gut, microbiota enzymatically modify bile acids. These modified bile acids are subsequently reabsorbed and returned to the liver. Approximately 5% of the bile acid pool escapes hepatic reuptake and enters the systemic circulation, thereby reaching extrahepatic tissues, including the ovary. Importantly, the ovary does not synthesize bile acids *de novo*; its tissue bile acids are entirely derived from circulating pools, which in turn depend on enterohepatic recycling. Thus, without an intact enterohepatic circulation, bile acids cannot reach the ovary.

Gut microbiota reshape bile acid composition through enzymatic activities such as bile salt hydrolase (BSH) and 7α-dehydroxylase. These modifications generate bioactive bile acids, including glycodeoxycholic acid (GDCA) and tauroursodeoxycholic acid (TUDCA). Such bile acids activate the Takeda G protein–coupled receptor 5 (TGR5) expressed on intestinal group 3 innate lymphoid cells (ILC3s), leading to activation of the GATA3 signaling pathway and enhanced secretion of interleukin-22 (IL-22). This step represents a critical signal conversion process, translating microbial–metabolic information into immune effector signaling directed toward distal organs.

In parallel, certain bile acids (e.g., GDCA) can directly bind TGR5 expressed in ovarian tissue, activating the cAMP/PKA signaling cascade. This pathway regulates mitochondrial function, mitigates oxidative stress, and modulates the expression of steroidogenic enzymes. Therefore, the ovary is not merely a passive recipient of immune-derived signals but also an active sensor of systemic bile acid–encoded metabolic information.

The signaling cascade of the gut–liver–ovary axis can be summarized as follows:

hepatic synthesis of primary bile acids → microbial modification to generate GDCA/TUDCA → maintenance of bile acid homeostasis through enterohepatic circulation → partial overflow into systemic circulation → activation of intestinal ILC3s via the TGR5–GATA3 pathway → IL-22 secretion → systemic transport of IL-22 to the ovary → direct activation of ovarian TGR5 by circulating bile acids → coordinated immune–metabolic regulation of the follicular microenvironment.

The designation “axis” reflects not a linear anatomical alignment but a closed-loop information network. Bile acids establish the physical circulation circuit; microbial enzymatic activity encodes metabolic signals; the TGR5–ILC3–IL-22 system converts these signals into immune mediators; and ovarian receptors function as distal decoding units. Together, these elements form an integrated regulatory system enabling remote modulation of ovarian physiology through gut microbiota–bile acid–immune interactions.

**Figure 1:**
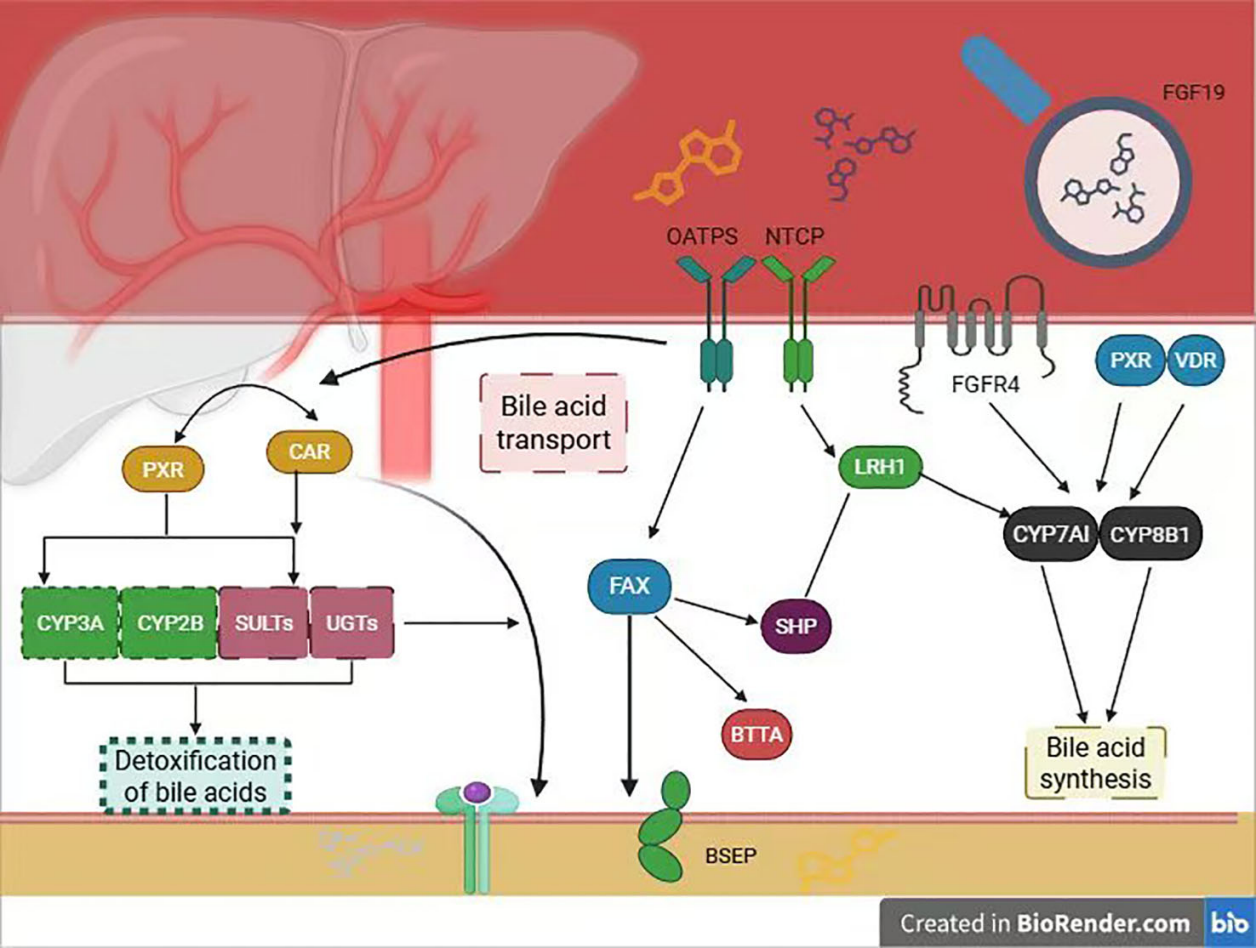
Bile acid metabolism mechanism.

**Figure 2:**
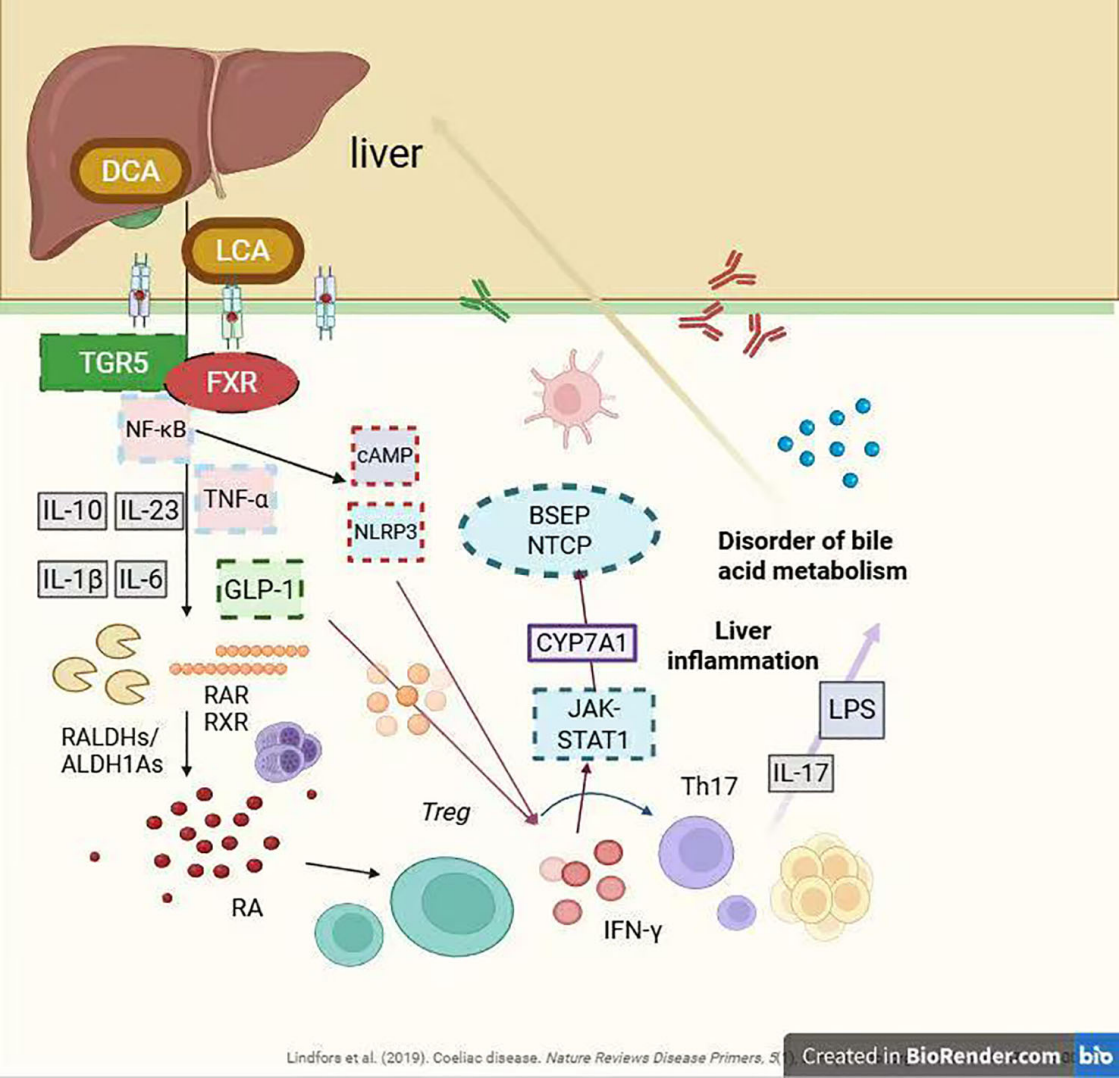
Interactions between immune cytokines and bile acid metabolism.

**Figure 3:**
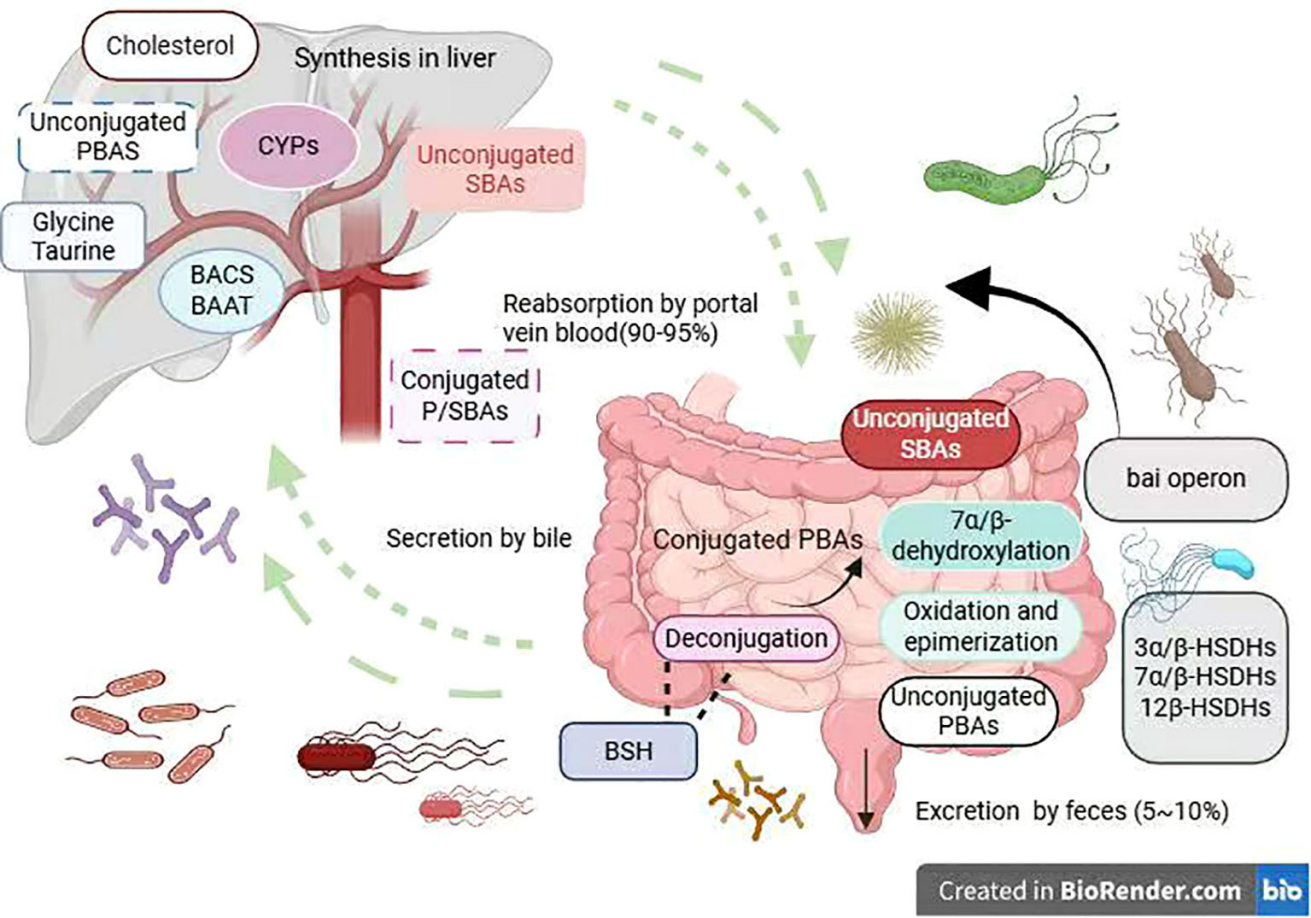
Gut–liver axis mechanism.

**Figure 4:**
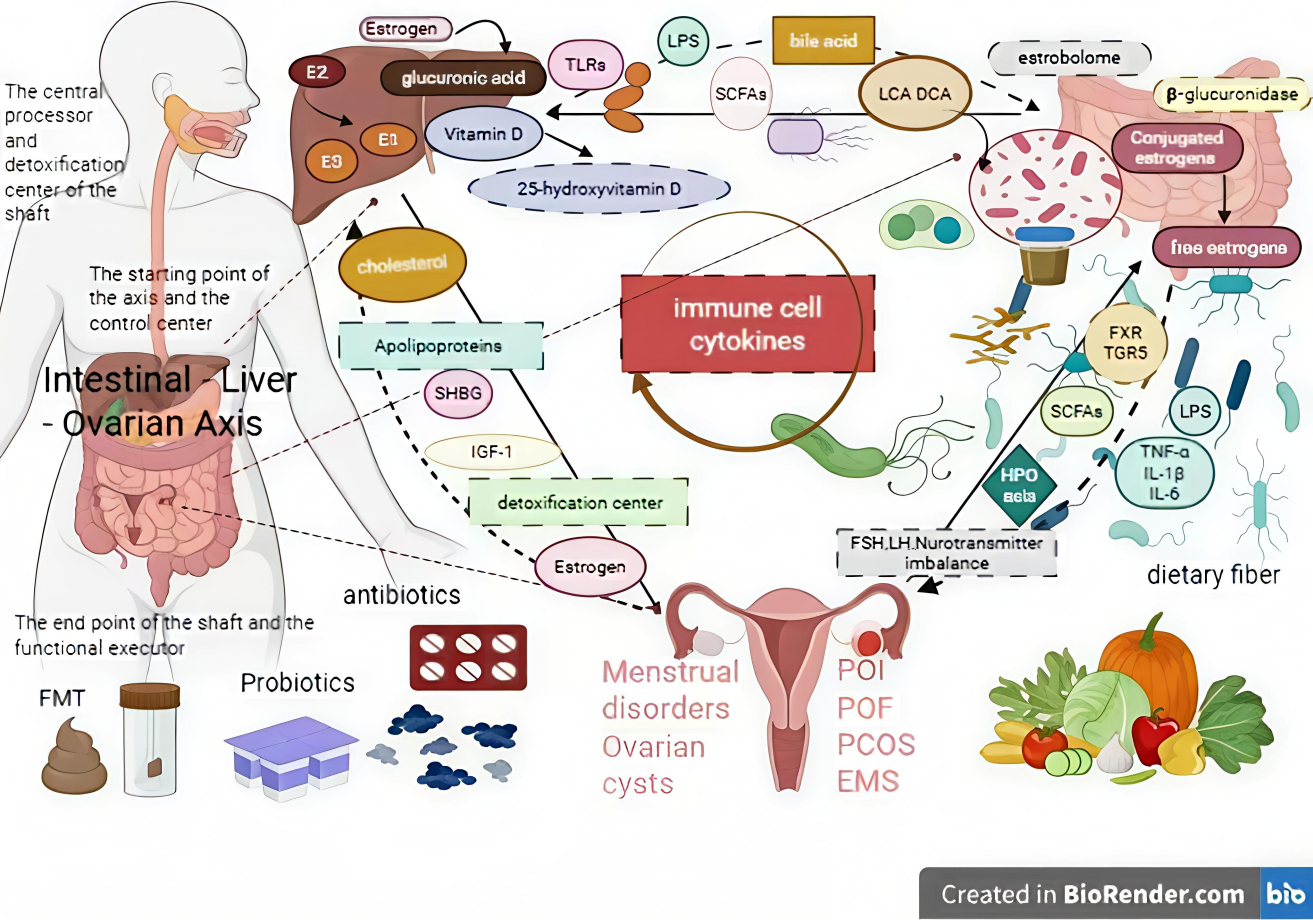
The link between immune cytokines, gut microbiota, bile acid metabolism, and premature ovarian failure (POF).

Current therapeutic strategies targeting this axis include inhibition of excessive BSH activity, supplementation with TUDCA, and enhancement of ILC3 function. These intervention nodes are considered among the most promising translational targets within the emerging field of microbiota-mediated reproductive regulation (**Figures 1**–**4**).

## 5 The role of growth hormone in the gut–liver–ovary axis

Growth hormone (GH) functions as a positive regulator at the liver–ovary interface of the gut–liver–ovary axis. Rather than directly modulating the upstream “microbiota–bile acid–ILC3” immune cascade, GH primarily exerts downstream regulatory effects by enhancing ovarian responsiveness to axis-derived signals.

The liver is among the most GH-sensitive organs. Upon binding of GH to the growth hormone receptor (GHR) on hepatocytes, the JAK2–STAT5 signaling pathway is activated, leading to transcriptional regulation of bile acid synthetic enzymes—such as CYP7A1 and CYP27A1—and bile acid transporters, including NTCP and BSEP.

Under physiological or replacement doses (0.2–0.5 mg/day), GH enhances hepatic bile acid synthetic capacity and improves enterohepatic circulation efficiency. Even when microbial bile acid modification is impaired (e.g., reduced GDCA production), the liver may compensate by increasing the output of bile acid precursors, partially restoring signaling molecule availability. Clinical studies indicate that adults with growth hormone deficiency (GHD) exhibit approximately a 50% reduction in hepatic bile acid uptake capacity, with significantly lower serum peak bile acid levels following oral administration compared with healthy controls. GH replacement therapy ameliorates hepatic steatosis, restores bile acid transporter expression, reduces the prevalence of nonalcoholic fatty liver disease (NAFLD) and cholestatic tendencies, and normalizes liver volume (64).

In addition, GH improves metabolic homeostasis by suppressing hepatic *de novo* lipogenesis (↓SREBP1c) and promoting fatty acid oxidation (↑PPARα).

In contrast, at supraphysiological or abusive doses (>1.0 mg/day), chronic GH exposure may induce hepatic insulin resistance, enhance gluconeogenesis, and promote lipid accumulation, resulting in nonalcoholic steatohepatitis (NASH)-like changes. Clinical case reports suggest that GH misuse in bodybuilding populations can lead to cholestatic liver injury, which is generally reversible upon discontinuation.

Within the ovary, both granulosa cells and oocytes express GHR and the bile acid receptor TGR5. GH transcriptionally upregulates ovarian TGR5 expression via the PKA–CREB signaling pathway, thereby enhancing ovarian sensitivity to bile acid signaling (65). Experimental studies have demonstrated that granulosa cells pretreated with GH exhibit increased STAT3 phosphorylation and elevated expression of IL-22 downstream antimicrobial peptides upon exposure to GDCA. These findings suggest that GH augments the efficiency of TGR5–IL-22 signal reception, indirectly optimizing immune modulation within the ovarian microenvironment.

Moreover, as a counter-regulatory hormone to insulin, GH at physiological doses promotes white adipose tissue browning and improves insulin sensitivity. This effect parallels the IL-22–mediated adipose browning pathway, and the two mechanisms may act synergistically to ameliorate hyperandrogenism in PCOS.

Collectively, GH enhances the overall functional efficiency of the gut–liver–ovary axis by maintaining hepatic bile acid transport capacity and strengthening ovarian responsiveness to TGR5-mediated signaling. Notably, the net hepatic effect of GH is strictly dose-dependent: physiological or replacement doses support hepatic homeostasis, whereas supraphysiological or abusive doses predispose to liver injury. This dose-dependent duality provides critical guidance for precision therapeutic strategies in reproductive endocrine disorders, including PCOS (66, 67).

## 6 Conclusion and future perspectives

Through systematic retrieval and analysis of both domestic and international databases, this review comprehensively evaluated the potential associations among immune cytokines, gut microbiota, bile acid metabolism, and premature ovarian failure (POF). The evidence indicates that these three components form a complex and interactive regulatory network that collectively contributes to the pathophysiology of POF through multiple mechanisms. POF has traditionally been attributed to genetic susceptibility, immune dysregulation, and iatrogenic injury. The introduction of the “gut–liver axis” concept provides a novel and systemic perspective, highlighting that the relationships among gut microbiota, hepatic metabolism, and ovarian function are not linear or unidirectional but instead involve dynamic bidirectional regulation centered on the integration of immunometabolic and endocrine signaling.

Gut dysbiosis and increased intestinal permeability (“leaky gut”) facilitate the translocation of bacterial endotoxins into the systemic circulation, triggering chronic low-grade inflammation. Persistent inflammatory stress in the liver further amplifies systemic cytokine production. These circulating inflammatory mediators reach the ovary, directly impair granulosa cell function, inhibit follicular development, and accelerate follicular atresia. This cascade illustrates how distal metabolic–immune disturbances may converge on ovarian dysfunction.

Future evaluation and management of POF are likely to expand into the microbiome dimension. From a diagnostic standpoint, integrative assessment may include fecal microbiota profiling—such as the abundance of short-chain fatty acid (SCFA)-producing bacteria and microbial diversity indices—combined with circulating inflammatory cytokine panels, bile acid profiling, and biomarkers of intestinal barrier integrity. Such a multidimensional framework could enable comprehensive evaluation of POF risk and immunometabolic status.

From a therapeutic perspective, microbiota-targeted interventions may become important adjunctive strategies. These include supplementation with specific functional strains capable of modulating Th17/Treg balance or producing SCFAs, as well as their corresponding prebiotics. In refractory cases, fecal microbiota transplantation may be explored to restore immune homeostasis. However, commercially available probiotics are primarily designed for gastrointestinal disorders (e.g., Lactobacillus, Bifidobacterium) and do not specifically target microbial signatures associated with PCOS or autoimmune POF, which typically include increased Bacteroides vulgatus, decreased Akkermansia muciniphila, and reduced butyrate-producing bacteria. Importantly, no currently available probiotic strain directly addresses the core pathogenic node of excessive BSH activity leading to GDCA/TUDCA deficiency.

Theoretically, targeted strategies such as low-BSH-activity B. vulgatus bacteriophages or engineered microbial strains may offer more precise intervention, yet this area remains largely unexplored. Targeted supplementation with A. muciniphila (available in certain international formulations) or butyrate-producing bacteria may assist in restoring intestinal barrier integrity, although rapid correction of bile acid composition should not be expected. Pharmacological targeting of bile acid signaling pathways—such as FXR or TGR5 agonists—and combination approaches integrating microbiota modulation with conventional hormone replacement therapy may facilitate a transition from symptomatic management toward mechanism-based treatment. Currently, however, accessible oral formulations of TUDCA or GDCA are limited, and ursodeoxycholic acid (UDCA), though clinically available, has comparatively weaker immunomodulatory effects.

Several potentially overlooked targets warrant attention. IL-22 represents the terminal effector molecule of the gut–liver–ovary axis. In animal models, direct IL-22 administration reverses ovarian pathology. Recombinant human IL-22 has progressed to phase II clinical trials in ulcerative colitis and graft-versus-host disease, yet no clinical exploration has been conducted in ovarian disorders. Targeting B. vulgatus constitutes an upstream intervention strategy; bacteriophage cocktail therapy has restored IL-22 levels and improved ovarian phenotypes in animal models, although clinical-grade phage preparations remain unavailable in many regions.

Dietary intervention may represent the most accessible and mechanistically supported strategy. Low-carbohydrate, high-fiber diets may reduce B. vulgatus abundance, increase A. muciniphila levels, and favor a higher proportion of conjugated bile acids. Despite its low cost and biological plausibility, dietary prescription remains underutilized in POF management. Promotion of high-fiber dietary patterns and healthy lifestyle modification may serve as foundational public health measures for preserving gut–liver axis integrity and reducing POF risk.

Notably, most advances in the gut–liver–ovary axis have focused on PCOS. Extending this framework to POF or endometriosis exposes several critical knowledge gaps. First, comprehensive ovarian receptor atlases are lacking. Single-cell transcriptomic data from reproductive-age ovaries do not yet provide a definitive reference map of bile acid receptor distribution across follicular stages and cell types. Second, the microbial–bile acid metabolic network remains incompletely characterized; the specific microbial contributors to DCA production or LCA depletion in PCOS have not been systematically defined, nor have dose–response relationships for pathogenic BSH activity been modeled. Third, the localization and functional consequences of IL-22 receptor (IL-22RA1) activation in ovarian tissue remain unclear. Whether IL-22–STAT3 signaling primarily suppresses inflammatory cytokines, induces antimicrobial peptides, or directly inhibits apoptosis requires further clarification beyond the general description of “inflammation reduction.”

Furthermore, substantial model mismatch exists: approximately 90% of mechanistic evidence derives from PCOS models, and extrapolation to POF may not be biologically equivalent. Dedicated POF-specific gut–liver–ovary axis models are urgently needed. Finally, key parameters for clinical translation—such as reference ranges for circulating bile acids, thresholds for ovarian dysfunction, and expected therapeutic response magnitudes—remain undefined.

The interaction between POF and the gut–liver axis exemplifies the principles of systems medicine: the ovary is not an isolated organ but is profoundly influenced by distal metabolic and immune states. Although this field remains in the stage of foundational research and early clinical exploration, it offers a compelling framework for understanding the complex etiology of POF. With continued investigation, gut–liver axis–targeted strategies may become an integral component of comprehensive POF management, ultimately providing new therapeutic opportunities for affected patients.
